# Decolorization of two synthetic dyes using the purified laccase of *Paraconiothyrium variabile* immobilized on porous silica beads

**DOI:** 10.1186/2052-336X-12-6

**Published:** 2014-01-06

**Authors:** Seyedeh-Shaghayegh Mirzadeh, Seyed-Mostafa Khezri, Shahla Rezaei, Hamid Forootanfar, Amir Hossein Mahvi, Mohammad Ali Faramarzi

**Affiliations:** 1 Department of Pharmaceutical Biotechnology, Faculty of Pharmacy and Biotechnology Research Center, Tehran University of Medical Sciences, Tehran 14176, Iran; 2 Department of Environmental Health Engineering, School of Public Health; Center for Solid Waste Research; Institute of Public Health Research, Tehran University of Medical Sciences, Tehran, Iran; 3 Department of Environmental Engineering, West Tehran Branch Islamic Azad University, Tehran, Iran; 4 Environment and Energy Department, Science and Research Branch, Islamic Azad University, Tehran, Iran; 5 Department of Pharmaceutical Biotechnology, Faculty of Pharmacy, Kerman University of Medical Sciences, Kerman, Iran; 6 Center for Water Quality Research, Institute for Environmental Research, Tehran University of Medical sciences, Tehran, Iran

**Keywords:** Laccase, Immobilization, Decolorization, Synthetic dye, 1-hydroxybenzotriazole

## Abstract

**Background:**

Decolorization of hazardous synthetic dyes using laccases in both free and immobilized form has gained attention during the last decades. The present study was designed to prepare immobilized laccase (purified from *Paraconiothyrium variabile*) on porous silica beads followed by evaluation of both free and immobilized laccases for decolorization of two synthetic dyes of Acid Blue 25 and Acid Orange 7. Effects of laccase concentration, pH and temperature alteration, and presence of 1-hydroxybenzotriazole (HBT) as laccase mediator on decolorization pattern were also studied. In addition, the kinetic parameters (*K*_*m*_ and *V*_*max*_) of the free and immobilized laccases for each synthetic dye were calculated.

**Results:**

Immobilized laccase represented higher temperature and pH stability compare to free one. 39% and 35% of Acid Blue 25 and Acid Orange 7 was decolorized, respectively after 65 min incubation in presence of the free laccase. In the case of immobilized laccase decolorization percent was found to be 76% and 64% for Acid Blue 25 and Acid Orange 7, respectively at the same time. Increasing of laccase activity enhanced decolorization percent using free and immobilized laccases. Relative decolorization of both applied dyes was increased after treatment by laccase-HBT system. After nine cycles of decolorization by immobilized laccase, 26% and 31% of relative activity were lost in the case of Acid Blue 25 and Acid Orange 7, respectively.

**Conclusions:**

To sum up, the present investigation introduced the immobilized laccase of *P*. *variabile* on porous beads as an efficient biocatalyst for decolorization of synthetic dyes.

## Introduction

Discharge of a wide range of synthetic dyes produced and being increasingly used by different industries to ecosystems made this subject as a major environmental problem [1,2]. In aquatic ecosystems, entrance of dyes decreases the oxygen level due to reducing the amount of sunlight for photosynthetic organisms [3]. Some of these dyes are toxic and carcinogen in nature and found to be recalcitrant [4]. Time and cost consuming characteristic of physicochemical methods like coagulation, flocculation, adsorption, ion-exchange, oxidation and electrochemical methods applied for removal of synthetic dyes [4,5] together with generating undesirable by-products prompts researchers to study on biological or enzymatic techniques for degradation of hazardous dyes [6,7]. Supplying much more eco-friendly process due to production of non-toxic metabolites, requiring mild conditions (temperature, pressure and pH) and less water consumption in comparison with physicochemical techniques and being cost-competitive are some advantages of enzymatic methods for decolorization and removal of synthetic dyes [1,3,5].

Laccases (benzenediol: oxygen oxidoreductases, EC 1.10.3.2) are multi-copper oxidases produced mainly by fungal strains, specially white-rot fungi [8,9]. The ability of laccases for oxidation of a broad range of substrates like chlorinated phenol, polycyclic aromatic hydrocarbons (PAHs), benzenethiols and textile dyes in presence of molecular oxygen as co-substrate introduced this biotechnologically important enzyme as the first choice for xenobiotic removal investigations [8,10]. However, low stability and low production yield are two main constraints for application of laccases in industrial processes [11,12]. Such mentioned problems could be overcome by development of immobilized laccases from different resources on solid supports which allow reusing of immobilized enzyme and may improve its stability [12,13]. Entrapment of enzyme in polymers, enzyme encapsulation in membranes, self-immobilization and covalent binding of enzymes to solid support are techniques used for fixation of enzymes [14] among which covalent biding supply more stable enzymes by increasing the rigidity of its structure and reducing protein unfolding [15]. During the recent years, wide range of supports like chitosan, chitosan/poly(vinyl alcohol) composite nanofibrous membranes, magnetic mesoporous silica nanoparticles and mesostructured siliceous cellular foams have been used for development of immobilized laccases [14]. Mesoporous silica materials are one of the most applied matrices for immobilization of enzymes due to specific properties such as hydrothermal resistance, well order porous structure and high surface area [16].

In the present study, the purified laccase from a soil isolated ascomycete, *P*. *variabile*, was immobilized on the porous silica particles and applied for decolorization of two synthetic dyes of Acid Orange 7 and Acid Blue 25 compared to free enzyme. Furthermore, the influences of conditional parameters such as enzyme activity, temperature, and pH on decolorization were investigated. The kinetic parameters (*K*_m_ and *V*_max_) of free and immobilized laccases through the applied dyes were also calculated.

## Materials and methods

### Enzyme, chemicals and dyes

The extracellular laccase of *P*. *variabile*, designated as *Pv*L, was purified from the culture broth of cultivated fungus using the method described by Forootanfar et al. [8] and used as enzyme source in the present study. CPC-silica beads pre-silanized with 3-aminopropyltriethoxysilane (APTES), glutaraldehyde solution (25%), and 2,2'-azinobis-(3-ethylbenzothiazoline-6-sulfonate) (ABTS) were purchased from Sigma-Aldrich (St. Louis, MO, USA). 1-Hydroxybenzotriazole (HBT) was provided by Merck Chemicals (Darmstadt, Germany). Two synthetic dyes, Acid Blue 25 (AB25) and Acid Orange 7 (AO7) (Table 1), were obtained from Alvan Sabet Co. (Tehran, Iran). All other reagents and chemicals were of the highest purity available.

**Table 1:** Chemical type, maximum absorbances, and kinetic parameters of two synthetic dyes

**Dye name**	**Type**	**λ**_**max **_**(nm)**	***K***_***m ***_**(mM)**	***V***_**max **_**(mmol min**^**-1**^ **mg**^**-1**^**)**
Acid orange 7	Azo	485	2.75^a^	146^a^
			2.25^b^	225^b^
Acid blue 25	AQ^c^	600	1.23^a^	165^a^
			0.736^b^	254^b^

^a^Calculated *K*_*m*_ and *V*_*max*_ in presence of immobilized laccase.

^b^Calculated *K*_*m*_ and *V*_*max*_ in presence of free laccase.

^c^Anthraquinone.

### Laccase immobilization

The *Pv*L was immobilized on the porous silica beads based on the method of Champagne and Ramsay [3]. Briefly, 4 g of pre-silanized CPC-silica beads (355–600 mm in diameter, an average surface area of 42.1 m^2^/g, and a pore size of 37.5 nm) was added to 2.5% glutaraldehyde solution (degassed under 2.0 bar vacuum pressure for 2 h) in 0.1 M KH_2_PO_4_ at pH 5.0 and stirred for 2 h. The enzyme solution (prepared in citrate buffer 0.1 M, pH 5) was then introduced to the activated silica beads and incubated at 4°C for 36 h. Consequently, the prepared beads was filtered through paper filter and washed three times with distilled water and twice with citrate buffer (0.1 M, pH 5.0). The SEM image of CPC-silica beads and immobilized *Pv*L on porous silica beads were then recorded using a scanning electron microscope (Philips XL30) operated at 5 kV.

### Determination of thermal and pH stability of free and immobilized *Pv*L

Stability of the prepared immobilized *Pv*L and the free purified laccase through temperature was studied by pre-incubating the free or immobilized *Pv*L at different temperatures between 20 and 60°C for 1 h, followed by determination of the residual activity. The influence of pH on the laccase stability was evaluated by incubating the free or immobilized *Pv*L at 4°C in different pH levels (3–8) for 24 h and determining the residual activity.

### Laccase activity determination

The laccase activity was measured by oxidizing the ABTS as a substrate according to the method previously described [17,18]. Briefly, 0.5 mL of the purified *Pv*L solution or 0.1 g of immobilized *Pv*L was added to 1.5 mL of the ABTS solution (5 mM in citrate buffer 0.1 M, pH 5) and incubated at 40°C and 120 rpm for 10 min. Change in the absorbance at 420 nm was then determined using a UV-vis spectrophotometer (UVD 2950, Labomed, Culver City, USA). The laccase activity was then calculated using the molar extinction coefficient of ABTS (ϵ_420_ = 36,000 M^-1^ cm^-1^). One unit enzyme activity was defined as the amount of enzyme requires oxidizing 1 μmol of ABTS per minute under the assay condition [19].

### Dye decolorization experiments

Decolorization experiments were performed by adding the free or immobilized *Pv*L (final activity of 2 U/mL) to each dye solution (a concentration of 400 mg/L achieved from a preliminary study), which was previously prepared by dissolving each dye in a citrate-phosphate buffer (0.1 M, pH 5.0), followed by incubation of the reaction mixture (final volume of 2.5 mL) at 40°C and 50 rpm for 1 h. Decolorization percentage was then determined by monitoring absorbance of the taken samples (each 5 min up to 1 h), using a UV-vis spectrophotometer at the maximum absorbance of each dye (Table 1). Incubation of the reaction mixture was then continued overnight to confirm any significant change in decolorization percentage. The following equation was used to estimate the percentage of decolorization:

$$\mathrm{Decolorization}\;\left(\%\right)=\left[A_{i}–A_{t}/A_{i}\right]\times 100$$

where A_i_ is the initial absorbance of the reaction mixture and A_t_ is the absorbance after incubation time [5,18]. In the case of free *Pv*L, the negative control was prepared by adding the heat-inactivated enzyme to the dye solution and incubating the reaction mixture as described above. In the case of immobilized *Pv*L, activated glass beads without enzyme applied as negative control. All experiments were performed in triplicate and means of decolorization percentages were reported.

### The effect of laccase activity on dye decolorization

In order to determine the effect of laccase activity on decolorization pattern, the purified or immobilized *Pv*L was added to the reaction mixture (as said above) to reach the enzyme activity of 0.5–8 U/mL followed by incubation at 40°C and 50 rpm for 60 min. The reaction mixture was then monitored for decolorization percentage. The final dye concentration in all experiments was 400 mg/L.

### The influence of pH on laccase-mediated decolorization

By adjusting the initial pH of each dye solution (final concentration of 400 mg/L) using 0.1 M citrate-phosphate buffer between 3 - 8, 2 U/mL free or immobilized *Pv*L was added to the reaction mixture and incubated at 40°C and 50 rpm for 60 min. The decolorization percentage was then monitored as previously described.

### The effect of temperature on enzymatic decolorization

The effect of temperature on enzymatic decolorization was studied by incubating 400 mg/L of the synthetic dyes in the presence of 2 U/mL of free or immobilized *Pv*L at temperature range of 25 - 70°C and pH 5.

### Application of laccase-HBT mediated system for dye decolorization

HBT as a non-phenolic laccase mediator was introduced to the reaction mixture (dye, 400 mg/L and free or immobilized *Pv*L, 2 U/mL in citrate buffer 0.1 M pH 5) to reach the final concentrations of 0.1, 1, and 5 mM. Decolorization was determined as previously described.

### Reusability of immobilized laccase

In order to determine the reusability of immobilized biocatalyst, the applied immobilized *Pv*L was recovered from the reaction mixture of each dye using filtration and washed three times with citrate buffer (0.1 M, pH 5). It was then introduced again to the reaction mixture and residual activity of the immobilized *Pv*L was measured as said above. The mentioned experiment was done in triplicate and mean of obtained results were reported.

### Kinetic studies

After determination of velocity in presence of different concentrations (100 - 600 mg/L) of each dye, the Michaelis - Menten curve was drawn by plotting the obtained velocity (V) against the dye concentrations (S). The kinetic parameters [*K*_m_ (Michaelis constant) and *V*_max_ (maximal velocity)] of the free or immobilized *Pv*L through each dye were then obtained using the Lineweaver - Burk transformation of the Michaelis - Menten equation.

### Statistical analysis

All above mentioned experiments performed in triplicate and the reported data were presented as mean ± standard deviation. The independent sample *t*-test and one-way analysis of variance (ANOVA) with Dunnett’s T3 post Hoc test was done using the SPSS 15 software (SPSS Inc) to calculate statistical significance between mean values. Probability values <0.05 were considered to be significant.

## Results and discussion

### Immobilization of laccase

**Figure 1:**
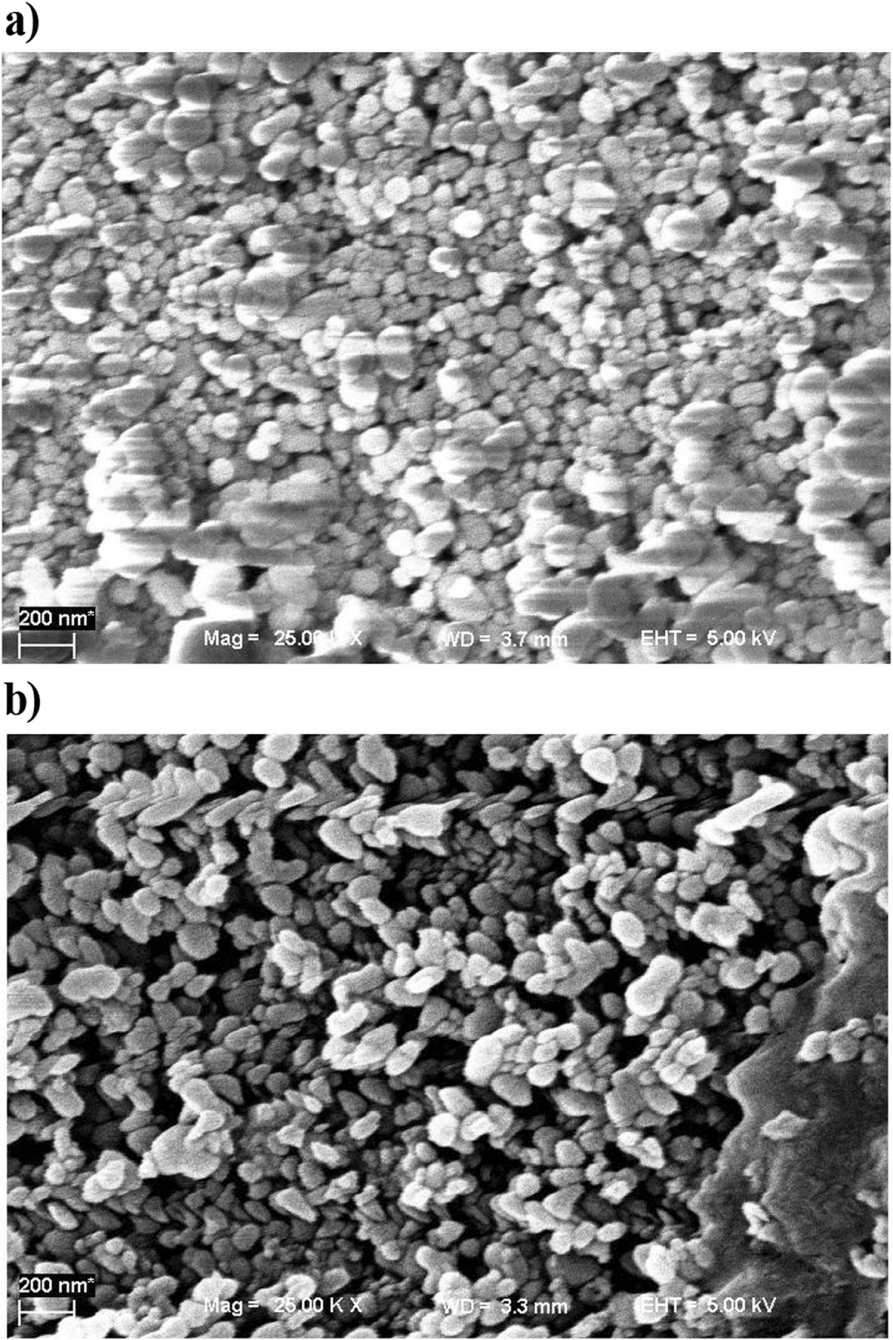
**SEM image. a**: CPC-silica beads; **b**: immobilized laccase on porous silica beads.

**Figure 2:**
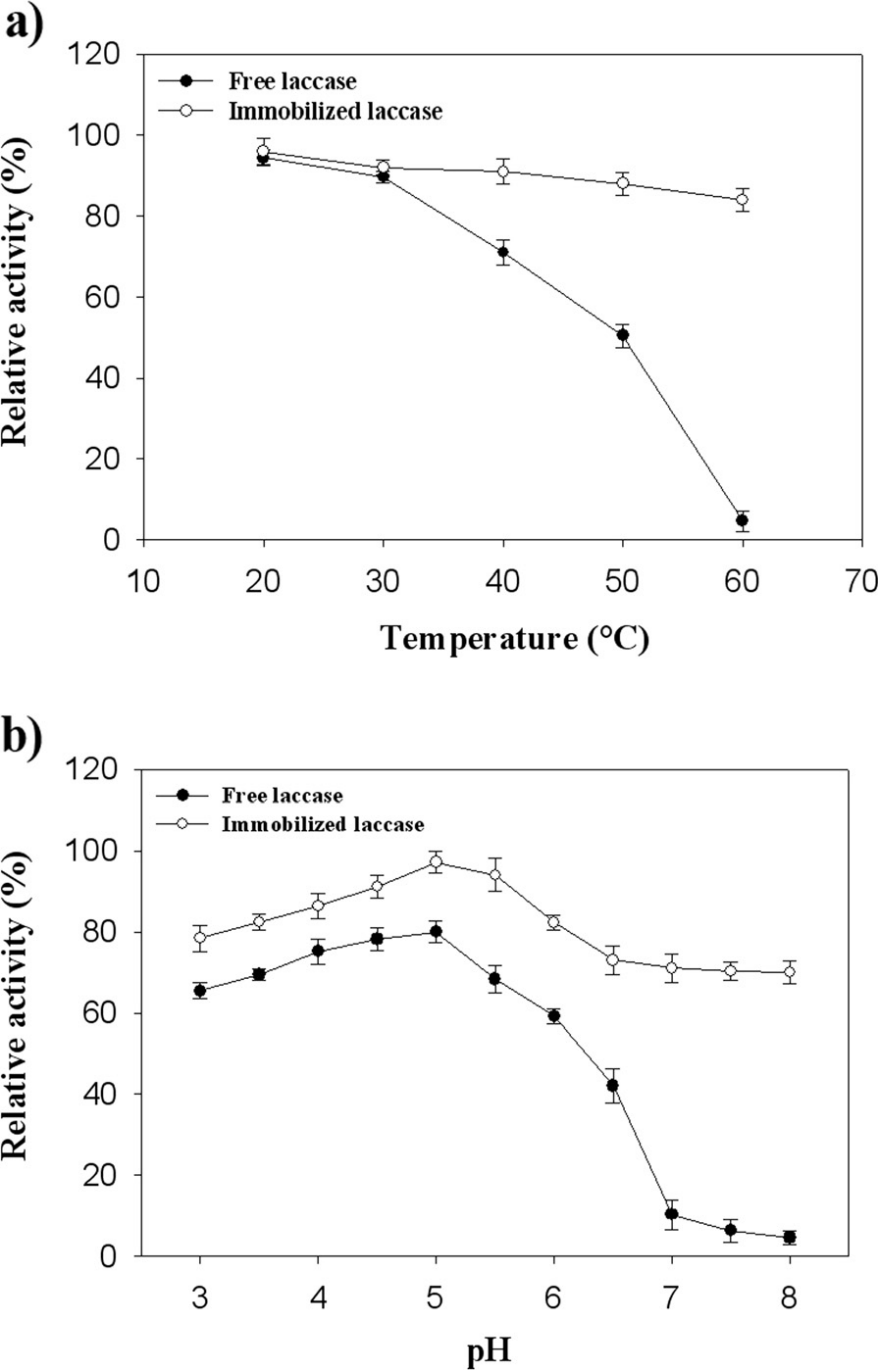
**Stability of the immobilized laccase. a**: Temperature stability; **b**: pH stability.

Figure 1a and Figure 1b represented the SEM images of CPC-silica beads and immobilized laccase on porous silica beads, respectively. Well dispersed particles without aggregation were formed after *Pv*L attachment (Figure 1b). It can be seen that smooth and spherical shape silica beads (Figure 1a) changed to rough and asymmetric particles after loading *Pv*L on silica beads (Figure 1b). Similar observation was reported by Sadighi and Faramarzi [18] after loading the laccase on chitosan nanoparticles. The enzymatic attachment was there introduced as the probable reason for such morphological modification. The nitrogen adsorption–desorption isotherm data before incorporation of the enzyme indicated that the surface area of silica is about 40 ± 5 m^2^/g (data not shown). Temperature and pH stability curve of the immobilized laccase compared to free enzyme are illustrated in Figure 2a and Figure 2b, respectively. At elevated temperature of 60°C the immobilized laccase showed 84% relative activity where only 4.6% initial activity of the free *Pv*L was remained at this temperature (Figure 2a). pH stability of the immobilized *Pv*L exhibited a broader range of activity in comparison with free laccase (Figure 2b).

Compare to free enzymes, immobilization of biocatalysts on solid supports made them more stable for industrial processes and allow them to be reused many times [14,20]. The obtained results of the present study revealed that immobilized laccase on porous silica beads represented higher temperature and pH stability compared to the free laccase. Same results were reported by Arica et al. [21] and Bayramoglu et al. [22] where the purified laccase of *Trametes versicolor* was immobilized on poly(GMA/EGDMA) and epoxy-functionalized magnetic chitosan beads, respectively. In the study of Wang et al. [23], it was revealed that immobilization of laccase on magnetic mesoporous silica particles enhanced the optimum catalytic temperature and pH of the enzyme from 20°C and pH 4 to 60°C and pH 5, respectively. Meanwhile, they showed that the immobilized laccase exhibited more than 95% of the maximum activity with a wider pH range between 4.0 and 5.5 [23].

### Decolorization using free and immobilized laccase

**Figure 3:**
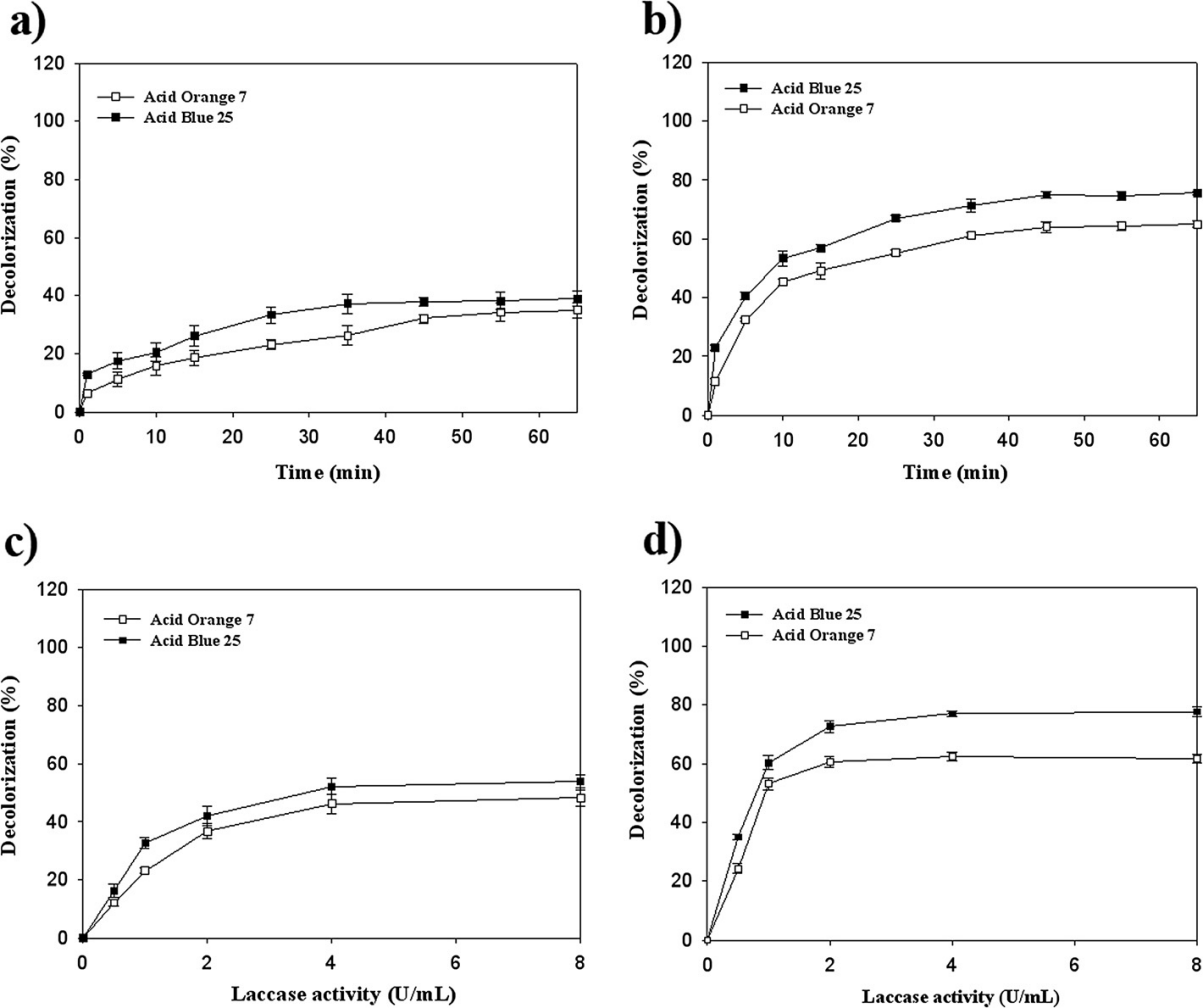
**Time course study and the influence of laccase activity on dye decolorization. a** and **c**: free laccase; **b** and **d**: immobilized laccase.

As shown in Figure 3a, the free *Pv*L (2 U/mL) was able to decolorize 39% of AB25 and 35% of AO7 after 65 min incubation while the immobilized enzyme removed 76% and 64% AB25 and AO7, respectively at the same time (Figure 3b). Further incubation of the reaction mixtures for 24 h did not show any significant increase in decolorization percentages (data not shown). In decolorization experiments, the chemical structure of applied dyes is one of the most important factors affects on decolorization percentage [1,24,25]. In general, azo dyes were found to be resistant to laccase-mediated decolorization while anthraquinone dyes are suitable substrate for laccases [1,4]. The obtained results of the present study showed higher decolorization percent for the anthraquinone dye of AB25 compare to azo dye of AO7 using both the free and immobilized laccases. In the study of Ashrafi et al. [26], where the free *Pv*L was applied for decolorization of thirteen synthetic dyes, the lowest decolorization percentage was achieved for Direct Blue 71 (a triazo dye) and Reactive Red 120 (a diazo dye) after 180 min incubation at 40°C.

### Effect of laccase activity on decolorization

Effect of increasing laccase concentration on decolorization of studied dyes was illustrated in Figure 3c and Figure 3d. In the case of free *Pv*L, the highest decolorization percent (52.2% for AB25 and 46.2% for AO7) was achieved at laccase activity of 4 U/mL (Figure 3c) while in the case of immobilized laccase maximum decolorization percent (75% for AB25 and 62% for AO7) was obtained in presence of 2 U/mL of laccase (Figure 3d). Depends on the sources of laccases (bacteria, fungi, etc.) decolorization pattern even on one special dye has been found to be different [4,27]. In our previous studies [4,7-10,18], the produced laccase of *P*. *varibile* was efficiently decolorized wide range of synthetic dyes including diazo dyes (Amido black, Sudan black, Congo red and Ponceau-S), triphenylmethane dyes (Crystal violet, Bromothymol blue, Commassie brilliant blue and Malachite green) and anthraquinone dyes (Rimazol brilliant blue R). The recent study of Ashrafi et al. [26] showed that increasing of laccase concentration from 0.025 to 0.1 U/mL significantly increased decolorization percent in all thirteen studied synthetic dyes.

### Influence of pH on decolorization

**Figure 4:**
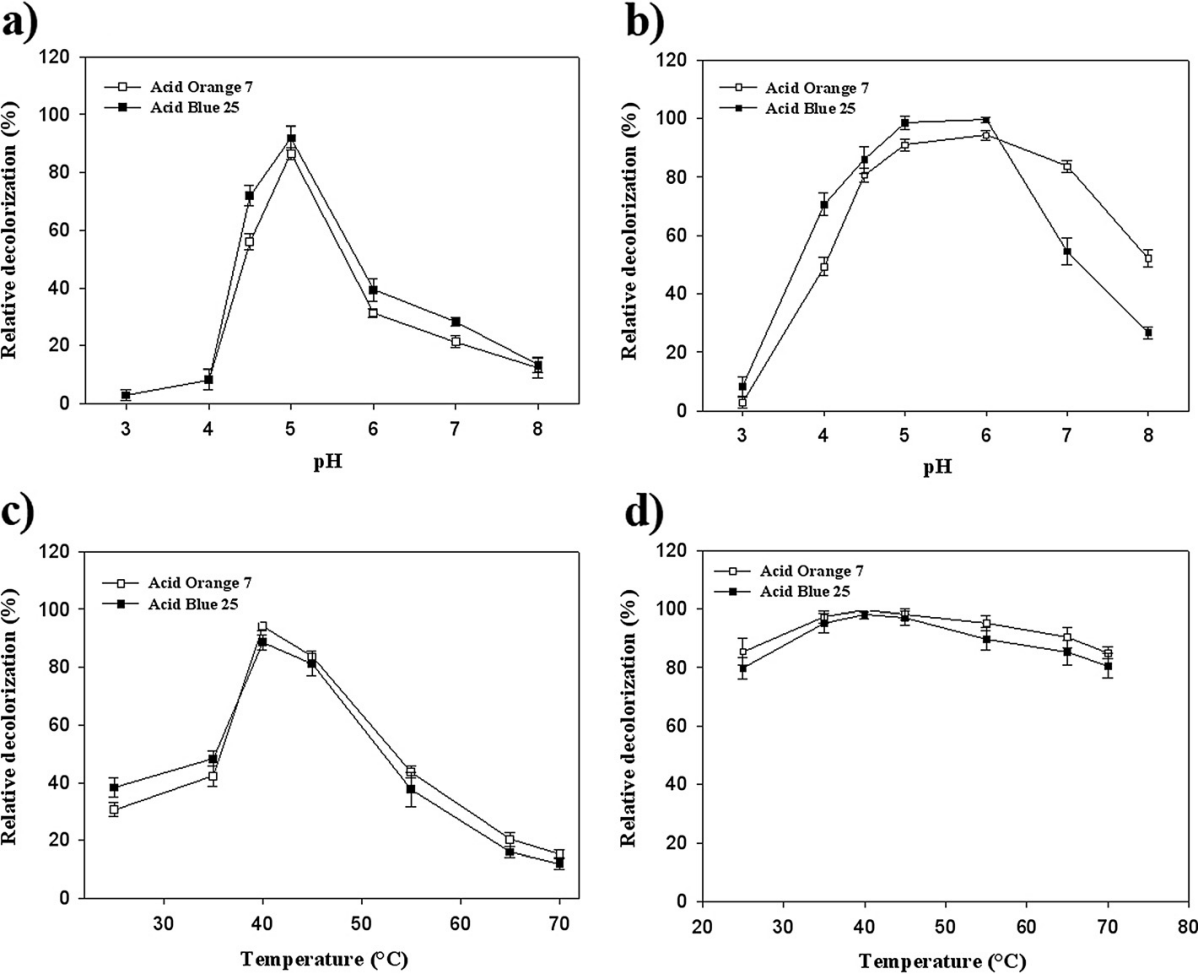
**Effects of pH and temperature on dye decolorization after 65 min incubation. a** and **c**: free laccase, **b** and **d**: immobilized laccase.

As represented in Figure 4a and Figure 4b the maximum decolorization in the case of both applied dyes and using free and immobilized laccases was occurred at optimum pH of *Pv*L (pH 5). Relative decolorization of free *Pv*L at pH 6 decreased by 35% and 31% in the case of AB25 and AO7, respectively (Figure 4a) while only 4% (AO7) and 1.5% (AB25) of maximum decolorization was lost in the case of immobilized laccase at pH 6 (Figure 4b). In comparison with the free laccase, the immobilized laccase exhibited more than 80% of the maximum decolorization with a wider pH range between 4.5 and 6 (Figure 4b).

### Effect of temperature on laccase decolorization

As shown in Figure 4c and Figure 4d the highest decolorization percent in the case of both synthetic dyes was obtained at optimum temperature of *Pv*L activity (40°C). At elevated temperature of 70°C, relative decolorization of both dyes was decreased below 20%. However, in the case of immobilized *Pv*L above 75% of initial decolorization remained at temperature of 70°C (Figure 4d). In the study of Sadighi et al. [18] it was found that the laccase immobilized on chitosan nanoparticles retained decolorization activity of Congo red at 95°C, and eventually lost 66% of decolorization activity at 100°C.

### Effect of HBT on laccase-mediated decolorization

**Figure 5:**
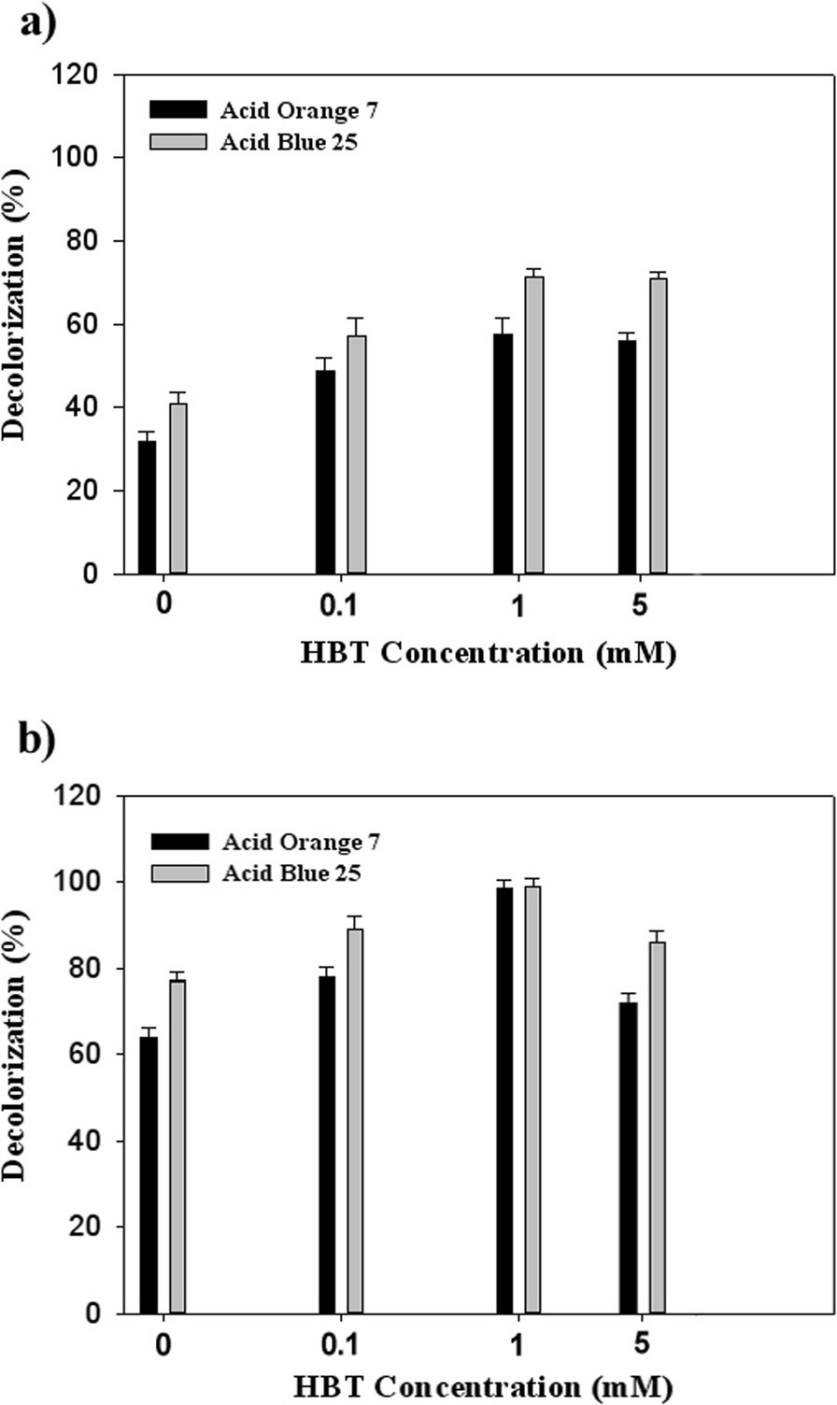
**Application of laccase-HBT system at a concentration of 0.1, 1, and 5 mM for dye decolorization. a**: free laccase; **b**: immobilized laccase.

Results of decolorization experiments using free and immobilized *Pv*L in absence and presence of HBT as laccase mediator are illustrated in Figure 5. In the case of free laccase, increasing of HBT concentration from 0.1 to 1 mM enhanced decolorization percent of both investigated dyes (Figure 5a). However, in the case of immobilized *Pv*L, higher concentration of HBT (above 5 mM) decreased decolorization percent (Figure 5b). Sadighi et al. [18] determined higher relative decolorization in presence of HBT (0.5 mM) using laccase immobilized on chitosan nanoparticles. Laccase-HBT system is one of the most successful laccase-mediated systems used for removal of pollutants, elimination of some synthetic dyes [5] and degradation of some pharmaceutical agents [19]. However, the mediating activity of HBT was found to be proportional due to the destructive effect of the N-O · group on the laccase activity, especially at a high concentration [28]. For example, decolorization percent of the triazo dye (Direct blue 71) was increased from 50% (unaided laccase) to 86% (in the presence HBT, 5 mM) using the free *Pv*L while inverse effect, decreasing decolorization percent from 97% (laccase alone) to 69.4% (laccase-HBT at a concentration of 5 mM), was observed in the case of Reactive Red 120 (a monoazo dye) [26].

### Reusability of immobilized laccase

**Figure 6:**
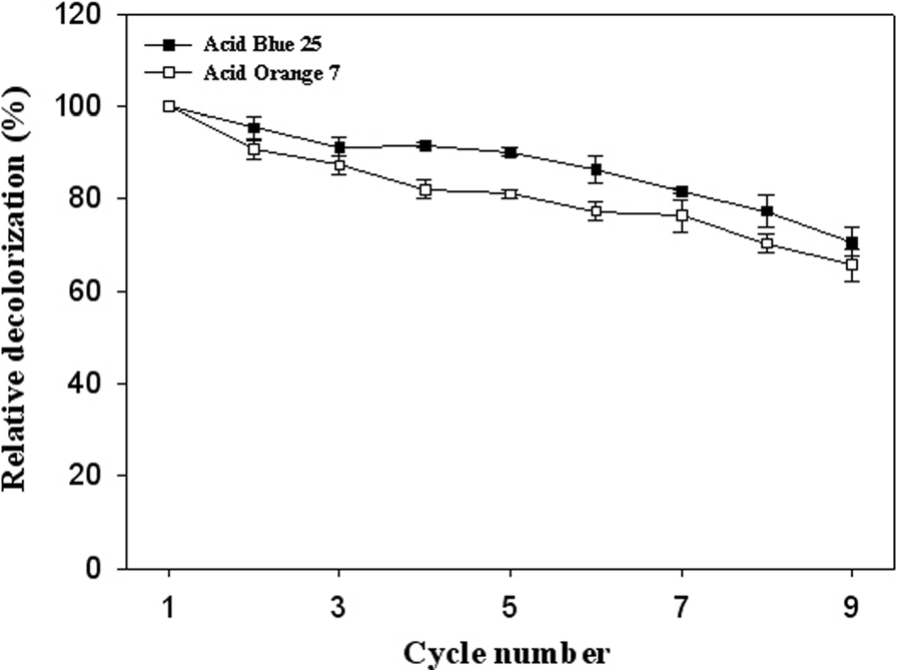
**Reusability potential of immobilized *Pv*L after 7 cycles of decolorization.** Data are presented as mean ± SD (n = 3).

The reusability of prepared immobilized enzymes is one of the most important factors for reducing overall cost of industrially applied enzymes [18,29,30]. The profile of decrease in relative decolorization of immobilized laccase during nine cycles of usage in our study is presented in Figure 6. Only 26% and 31% initial activity of the immobilized *Pv*L lost after nine cycles of application in the case of AB25 and AO7, respectively. The results of our previous study revealed that relative decolorization of *P*. *variabile* laccase immobilized on alginate-gelatin mixture fallen to 20% when applied seven times in decolorization experiments [7]. However, study of Sadighi et al. [18] showed that 96% of biocatalytic (the laccase from *Aspergillus oryzae*, Denilite IIS, immobilized on glass beads) activity was still retained after 25 reusing rounds of Congo red decolorization. About 60% initial activity of the immobilized laccase of *Pleurotus sajor*-*caju* on functionalized SBA-15 mesoporous silica was remained after fourteen reaction cycles [11].

### Kinetic parameters of free and immobilized laccases

Kinetic parameters of free and immobilized laccases toward two studied dyes of AB25 and AO7 were summarized in Table 1. Since anthraquinoid dyes especially those with amine groups on the ring are more suitable substrates than azo dyes for laccase [31], *K*_*m*_ value of both free and immobilized laccases for AB25 is lower than AO7 and also the degradation of AB25 is faster than AO7, i.e., the *V*_max_ for AB25 is higher than AO7. On the other hand, the higher *K*_*m*_ value of immobilized *Pv*L toward both applied dyes means lower affinity of immobilized laccase for applied dyes compared to free laccase. The obtained results are in agreement with the results achieved by Rekuc et al. [32] indicating 3.4 fold increase in *K*_*m*_ value of immobilized laccase compared to free laccase. Such observation might be due to structural change in the adsorption process of laccase onto solid support and low accessibility of the substrate to the active site of the immobilized laccase [23,33].

## Conclusion

The purified laccase of *P*. *variabile*, recently studied for its capacity in the removal of aromatic compounds (e.g., chlorophenol pollutants, hazardous synthetic dyes, and pharmaceutical agents), was immobilized on porous silica beads and applied for decolorization of two synthetic dyes of Acid Blue 25 (an anthraquinone dye) and Acid Orange 7 (an azo dye). pH and thermal stability of immobilized enzyme was improved compare to free laccase. Both applied dyes were efficiently decolorized using immobilized laccase. Application of the laccase-HBT system improved the decolorization of both synthetic dyes using free and immobilized laccases.

## Competing interests

The authors declare that they have no competing interests.

## Authors’ contributions

SSM carried out decolorization studies. Production and purification of laccase from *P*. *variabile* culture broth was performed by HF. SMK participated in decolorization studies and writing of the manuscript. SR participated in the studies of decolorization kinetic. AHM contributed in writing of the manuscript, decolorization studies and analyzing of data. MAF involved in purchasing of required materials and instruments, designing of decolorization experiments, analyzing of data and reviewing of the manuscript. All authors read and approved the final manuscript.
